# A Novel MIMO-SAR System Based on Simultaneous Digital Beam Forming of Both Transceiver and Receiver

**DOI:** 10.3390/s20226604

**Published:** 2020-11-18

**Authors:** Yuzhen Zhao, Longyong Chen, Fubo Zhang, Yanlei Li, Yirong Wu

**Affiliations:** 1 Aerospace Information Research Institute, Chinese Academy of Sciences, Beijing 100190, China; zyz309@mail.ustc.edu.cn (Y.Z.); fbzhang@mail.ie.ac.cn (F.Z.); yllee@mail.ie.ac.cn (Y.L.); wyr@mail.ie.ac.cn (Y.W.); 2 National Key Laboratory of Microwave Imaging Technology, Beijing 100190, China; 3 School of Electronics, Electrical and Communication Engineering, University of Chinese Academy of Sciences, Beijing 100049, China

**Keywords:** multi-input multi-output synthetic aperture radar (MIMO-SAR), orthogonal frequency division multiplexing chirp (OFDM chirp), digital beamforming (DBF)

## Abstract

Orthogonal frequency division multiplexing (OFDM) chirp waveform, which is composed of two or more successive identical linear frequency modulated sub pulses, is a newly proposed orthogonal waveform scheme for multi-input multi-output (MIMO) synthetic aperture radar (SAR) systems. However, according to the waveform model, there will be range ambiguity if the mapping width exceeds the maximum unambiguous width determined by the transmitted signal. This greatly limits its application in high-resolution wide-swath (HRWS) remote sensing. The traditional system divides the echoes by digital beam forming (DBF) to solve this problem, but the energy utilization rate is low. A MIMO-SAR system using simultaneous digital beam forming of both transceiver and receiver to avoid range ambiguity is designed in this paper. Compared with traditional system, the novel system designed in this paper obtain higher energy utilization and waveform orthogonality.

## 1. Introduction

Synthetic aperture radar (SAR), as an all-time all-weather, high-resolution active microwave imaging radar, has unmatched advantages over visible light and infrared detection systems. It has strengths in military reconnaissance, disaster monitoring and terrain measurement. It has been extensively used in many fields such as surveying, mapping, marine research and resource exploration. The basic principle is to use the relative movement between the azimuth antenna and the target for virtually synthesize a large aperture, so as to obtain higher azimuth resolution [1].

For the traditional SAR, to increase the mapping width, it is necessary to reduce the aperture of the antenna. The reduction of the antenna aperture will reduce the gain of the antenna, making the power aperture product smaller and the imaging capability worse. In addition, high azimuth resolution requires SAR systems to have high pulse repetition frequency. High pulse repetition frequency will lead to the increase of pulse blind area, which further limits the mapping width. Therefore, single-channel SAR and single-transmit and multiple-receive (SIMO) SAR cannot satisfy the growing demand for mapping. Multiple-input multiple-output synthetic aperture radar (MIMO-SAR) provides more channels and large aperture of the antenna [2]. So, MIMO-SAR can meet the needs of both increasing the aperture of the antenna and maintaining a large mapping width. In addition, multiple azimuth equivalent phase centers can improve the Doppler resolution of the system, thereby relaxing the limit of pulse repetition frequency [3,4]. MIMO-SAR systems used for 3-D imaging, high-resolution wide-swath (HRWS) remote sensing and multi-baseline interferometry have received considerable attention in recent years [5,6,7,8,9]. These applications are severely limited by the available sets of orthogonal waveforms [10]. On one hand, the waveform of SAR scheme should have a large bandwidth in order to obtain high range resolution. On the other hand, the signal in different channel must be orthogonal. How to conceive of orthogonal waveforms is a research hotspot in recent years. Next, we will provide a brief introduction to the orthogonal waveforms used in MIMO-SAR.

In MIMO communication and MIMO radar, code division signal is widely used as an orthogonal signal, and has achieved good results. Therefore, scholars have been conducted research on the application of code division signal in MIMO-SAR. Linear frequency modulation (LFM) signal has the advantages of large time-width bandwidth product and ideal correlation performance. It is the most commonly used signal in SAR. Up and down chirp signals are a pair of code division signals. Therefore, MIMO-SAR mostly uses up and down chirp signals as the basic form, combining different modulation methods to form code division signals [11]. Regardless of the combination of modulation methods, the common problem of code division signals is that the blurred energy is not removed from the image, but is scattered in the range and time domain. MIMO-SAR observes distributed scenes. The blur energy of different targets will generate vector additions to make the integrated sidelobe level ratio (ISLR) greatly deteriorates. It also influences the signal to interference plus noise ratio (SINR) of the image, and seriously reduces the image quality. It is particularly obvious in larger scene applications [12,13,14,15]. Therefore, code division signals could not be used independently as MIMO-SAR orthogonal signals [10].

Since the cross-correlation between the frequency division signals is 0, some scholars use them in MIMO-SAR systems. Different antennas are used to transmit LFM signals with stepped center frequencies. The echoes transmitted by different antenna can be separate by band-pass filtering. Subsequently, the separated echoes are rearranged in the azimuth direction to increase the number of spatial samples, thereby improving the azimuth resolution of the system with the same pulse repeat frequency (PRF). However, the above method causes different signal frequencies at different azimuth positions, which will inevitably lead to changes in the scattering characteristics of the target. It will result in a reduction in imaging quality. Some scholars proposed a multi-sub-band concurrent MIMO-SAR HRWS imaging method, which uses multiple antennas to transmit and receive stepped frequency signals at the same time, uses synthesis in time domain to synthesize the received echoes of sub-bands in the range direction [16]. Combined with azimuth de-blurring processing, HRWS imaging can be realized. This method has the same signal center frequency and bandwidth at all azimuth sampling points and is no longer restricted in high-resolution applications. In practical system applications, this method is susceptible to the non-ideal roll-off characteristics of the filter. The spectrum of each sub-band overlaps after band-pass filtering. It will raise the sidelobe level of the sub-band synthesis result, and even generate a grating. The flap reduces the image quality, so higher requirements are placed on the system design.

Space time coding (STC) signals introduce time and space correlations in the signals transmitted by different antennas and improve the diversity gain and coding gain of the system without increasing the bandwidth. STC signals have been widely used in wireless communications [17]. Some scholars use separate dual antennas to transmit coded signals in turn, and use the space-time two-dimensional orthogonality of the signals to combine the echoes received by multiple antennas at adjacent pulse repetition time (PRT) to achieve STC, reconstruct the SAR image corresponding to each pair of transmitting and receiving antennas. STC signals are theoretically orthogonal to each other. It can also obtain space diversity gain and array gain, improve the performance of the SAR system. However, the premise is that the channel has time-invariant characteristics. Some scholars put forward some improvement methods in [18,19,20]. STC signals are more suitable for airborne SAR where the PRF can be far larger than the Doppler bandwidth.

The azimuth phase modulation signal mainly includes azimuth phase coding (APC) signals and inter-pulse phase modulation signals [21,22]. Azimuth phase encoding was first used to suppress range ambiguity in single-channel spaceborne SAR. Later, scholars gave a SAR imaging process based on azimuth phase encoding to suppress range ambiguity, and used it to break through the limitation of the minimum aperture of antenna. The application prospects of the method in single-channel SAR and SIMO SAR [23]. Some scholars combine digital beamforming technology with azimuth phase encoding and apply them to SIMO SAR HRWS imaging [24,25]. Based on similar principles, scholars have proposed a method of generating MIMO-SAR orthogonal signals using inter-pulse phase modulation. This method modulates the transmitted signals of different channels to different Doppler center frequencies. The above two azimuth phase modulation methods use the weighting of the azimuth two-way antenna pattern to achieve the suppression of ambiguity energy, and separate multiple signal echoes through band-pass filtering in the Doppler frequency domain or azimuth digital beam forming (DBF) [26]. The system PRF can ensure that when the azimuth spectrum of each orthogonal signal does not overlap, it can effectively isolate the energy of different signals. The premise of this method is that the system has a higher oversampling rate in the azimuth direction. The PRF need to be greater than the sum of the Doppler bandwidth of all echoes.

Orthogonal frequency division multiplexing (OFDM) is the waveform used in 4G/5G communication. It has large bandwidth and great orthogonality, so there are a lot of researches on the combination of OFDM and MIMO-SAR in recent years [27,28,29,30,31]. One of them is to modulate the spectrum of LFM signal to different subcarriers to form two orthogonal and constant modulus signals. Some scholars call it interleaved OFDM, also known as OFDM LFM signal or OFDM chirp signal. Some scholars improved it to avoid the small frequency offset needed in the signal generation and ensure the good orthogonality of the signal [32]. Co-frequency orthogonal signals mainly refer to OFDM chirp signals, and short-term shift-orthogonal (STSO) signals [6,7,32]. In little time range, the cross-correlation of the STSO signals is 0. For the echo outside the time range which cannot be separated by the orthogonality of the signal, it is necessary to combine DBF technology to form a narrow beam to suppress it. Similarly, OFDM chirp signals also need DBF to impress range ambiguity. OFDM chirp signal is identical with chirp signal after demodulation, so it has excellent performance in ISLR and peak side lobe ratio (PSLR). This is also the reason why OFDM chirp signal is chosen as the main research object in this paper. The work of this paper is to improve the MIMO-SAR system while retaining the advantages of OFDM chirp signal.

The OFDM chirp signal performs well in both orthogonality and range resolution, but there is an inherent range ambiguity due to modulation and demodulation methods [32]. The traditional method uses DBF for received echo to suppress the inherent range ambiguity. However, in order to obtain a wide beam, the transmitter uses a small antenna aperture. This means that the antenna gain is relatively low. To improve this situation, this paper proposes a MIMO-SAR system based on simultaneous digital beam forming of both transceiver and receiver. Compared with the traditional method, the proposed method uses DBF technology in the transmitting antenna, so it can use a large antenna aperture to obtain higher gain. Plus, in the range direction, in order to further suppress the range ambiguity, frequency diversity technology is used. It can be seen from the simulation analysis that the method proposed in this paper can improve the signal-to-noise ratio (SNR) and has a better effect of range ambiguity suppression.

The remainder of this paper is organized as follows: The MIMO-SAR system model which uses OFDM chirp signal is presented in Section 2. The principle of the method proposed in this paper is elaborated in Section 3. In Section 4, theoretical analysis and simulation results illustrate the feasibility of this system. Finally, in Section 5, a discussion of this work is provided.

## 2. MIMO-SAR System Model Uses OFDM Chirp Signal

In this section, the MIMO-SAR model is first introduced, taking four transmitters and four receivers as an example. Then, several typical orthogonal waveforms are introduced. Their advantages and disadvantages are analyzed, respectively. Finally, the reason for the range ambiguity of OFDM chirp signal is analyzed theoretically.

### 2.1. MIMO-SAR System

**Figure 1:**
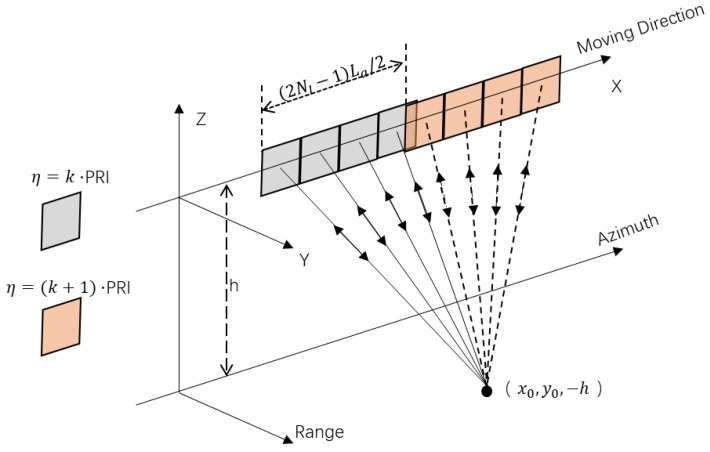
Geometry of multiple-input multiple-output synthetic aperture radar (MIMO-SAR).

As shown in Figure 1, the MIMO-SAR system has four antennas to transmit orthogonal waveforms, and all of its four antennas are used for receiving echoes. The platform equipped with regularly and compactly spaced antennas is required to move $\left(2N_{l}-1\right)\;L_{a}/2$ during one pulse repeat interval, where $L_{a}$ denotes the antenna length and $N_{l}$ is the subantenna number. It is necessary to correct the result if this restriction is not fulfilled [33]. There are $2N_{l}-1$ effective equivalent phase centers in the system at each slow time. The equivalent PRF of the system is $2N_{l}-1$ times of the actual PRF.

The platform moves at velocity *V* in the azimuth direction, and the origin locates at the center of the antenna array at the slow time *η* = 0. The coordinate of ground point target T is $\left(x_{0},\;y_{0},\;-h\right)$. For the signal transmitted from the pth ($p=1,\ldots ,N_{l}$) antenna and received by the qth ($q=1,\ldots ,N_{l}$) antenna, the range history is

(1) $$\begin{array}{cc} R_{p,q}\left(\eta \right) & =R_{p}\left(\eta \right)+R_{q}\left(\eta \right)\\ & =\sqrt{{\left(x_{0}-V\eta -\left(p-\left(N_{p}+1\right)/2\right)L_{a}\right)}^{2}+y_{0}{}^{2}+h^{2}}\\ & +\sqrt{{\left(x_{0}-V\eta -\left(q-\left(N_{p}+1\right)/2\right)L_{a}\right)}^{2}+y_{0}{}^{2}+h^{2}} \end{array}$$

where $R_{p}\left(\eta \right)$ denotes the range history between the pth antenna and the target. Compared with single channel SAR, MIMO-SAR needs to rearrange the received echo in the azimuth direction, so as to obtain the echo with high PRF equivalent to single channel SAR.

### 2.2. OFDM Chirp Signal and the Reason of Range Ambiguity

Through the above analysis and discussions, it can be found that each waveform has its pros and cons. As an orthogonal waveform, the OFDM chirp waveform has been proven to be effective. In this part, the principle of OFDM chirp signal and the cause of range ambiguity will be introduced.

The chirp signal is modulated to different carrier frequencies of the OFDM signal. Two-channel improved OFDM chirp signals have been proposed in [8]. In this paper, we get M-channel orthogonal OFDM chirp signals, where M is any integer, their time domain is as follows:

(2) $$S=[\begin{array}{ccccc} s & s & s & \ldots & s\\ s\cdot e^{1\cdot \frac{0\cdot j2\pi }{M}} & s\cdot e^{1\cdot \frac{1\cdot j2\pi }{M}} & \ldots & & s\cdot e^{1\cdot \frac{\left(M-1\right)\cdot j2\pi }{M}}\\ s\cdot e^{2\cdot \frac{0\cdot j2\pi }{M}} & s\cdot e^{2\cdot \frac{1\cdot j2\pi }{M}} & \ldots & & s\cdot e^{2\cdot \frac{\left(M-1\right)\cdot j2\pi }{M}}\\ \ldots & \ldots & & \ldots & \ldots \\ s\cdot e^{\left(M-1\right)\cdot \frac{0\cdot j2\pi }{M}} & \ldots & & & s\cdot e^{\left(M-1\right)\cdot \frac{\left(M-1\right)\cdot j2\pi }{M}} \end{array}]$$

for $s=\exp\left(j\pi k_{r}t^{2}\right)$, t∈[0,T], T denotes the pulse length of the modulated chirp signal and $k_{r}$ denotes the frequency modulation. S denotes transmission signal, each row of the matrix corresponds to each orthogonal signal.

Transform (2) into frequency domain and we get:

(3) $$\begin{array}{cc} X_{1}\left(Mk+1\right) & =\mathrm{DFT}[\exp\left(\;j\pi k_{r}{\left(\frac{k-1}{F_{s}}\right)}^{2}\right)],X_{1}\left(p|p\neq Mk+1\right)=0\\ X_{2}\left(Mk+2\right) & =\text{DFT}\left[\exp\left(\;j\pi k_{r}{\left(\frac{k-1}{F_{s}}\right)}^{2}\right)\cdot \exp\left(-j\frac{2\pi }{MN\left(k-1\right)}\right)\right],X_{2}\left(p|p\neq Mk+2\right)=0\\ X_{3}\left(Mk+3\right) & =\text{DFT}\left[\exp\left(\;j\pi k_{r}{\left(\frac{k-1}{F_{s}}\right)}^{2}\right)\cdot \exp\left(-j\frac{2\pi }{MN}\cdot 2\left(k-1\right)\right)\right],X_{3}\left(p|p\neq Mk+3\right)=0\\ & .\\ & .\\ & .\\ X_{M}\left(Mk+M\right) & =\text{DFT}\left[\exp\left(\;j\pi k_{r}{\left(\frac{k-1}{F_{s}}\right)}^{2}\right)\cdot \exp\left(-j\frac{2\pi }{MN}\cdot \left(M-1\right)\left(k-1\right)\right)\right],X_{M}\left(p|p\neq Mk\right)=0 \end{array}$$

for k=0,1,2,…,N−1, p=1,2,…,MN, $F_{s}$ denotes the sampling rate, the DFT length is N and $N=F_{s}\cdot T$.

It can be seen from (3), the signal from each group of subcarriers is equal to the chirp signal multiplied by a carrier term. After demodulation, a result equivalent to the chirp signal can be obtained after processing each group of sub-carriers.

**Figure 2:**
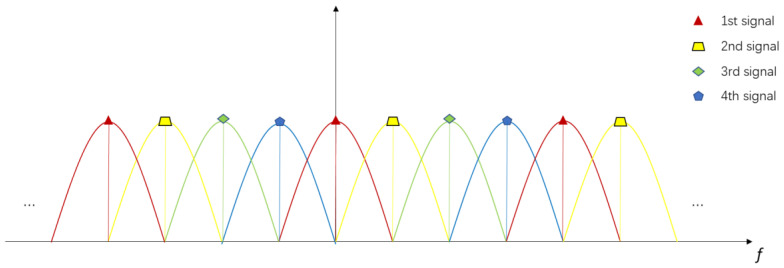
Orthogonal frequency division multiplexing (OFDM) chirp signal in frequency domain.

Taking the four-channel signals as an example, the modulation method is as shown in Figure 2:

The chirp signal is separately modulated into four different subcarriers in frequency domain, and then transmitted through the transmitting antenna, respectively. As all the subcarrier frequencies are orthogonal, each chirp signal can be demodulated by extracting the weight of its own subcarrier frequencies from the combination of the four OFDM waveforms.

In theory, since each subcarrier is orthogonal, this coding method can obtain four sets of orthogonal signals. However, because of the information is not fully utilized during demodulation, range ambiguity occurs.

Equation (4) is the form of OFDM chirp signal in time domain:

(4) $$\begin{array}{cc} x_{1}\left(t\right) & =\text{rect}\left(\frac{t}{T}\right)\cdot \exp\left(j\pi k_{r}t^{2}\right)+\text{rect}\left(\frac{t-T}{T}\right)\cdot \exp\left(j\pi k_{r}t^{2}\right)\\ & +\text{rect}\left(\frac{t-2T}{T}\right)\cdot \exp\left(j\pi k_{r}t^{2}\right)+\text{rect}\left(\frac{t-3T}{T}\right)\cdot \exp\left(j\pi k_{r}t^{2}\right)\\ x_{2}\left(t\right) & =\text{rect}\left(\frac{t}{T}\right)\cdot \exp\left(j\pi k_{r}t^{2}\right)+\text{rect}\left(\frac{t-T}{T}\right)\cdot \exp\left(j\pi k_{r}t^{2}+\frac{j\pi }{2}\right)\\ & +\text{rect}\left(\frac{t-2T}{T}\right)\cdot \exp\left(j\pi k_{r}t^{2}+j\pi \right)+\text{rect}\left(\frac{t-3T}{T}\right)\cdot \exp\left(j\pi k_{r}t^{2}+\frac{3j\pi }{2}\right)\\ x_{3}\left(t\right) & =\text{rect}\left(\frac{t}{T}\right)\cdot \exp\left(j\pi k_{r}t^{2}\right)+\text{rect}\left(\frac{t-T}{T}\right)\cdot \exp\left(j\pi k_{r}t^{2}+j\pi \right)\\ & +\text{rect}\left(\frac{t-2T}{T}\right)\cdot \exp\left(j\pi k_{r}t^{2}\right)+\text{rect}\left(\frac{t-3T}{T}\right)\cdot \exp\left(j\pi k_{r}t^{2}+j\pi \right)\\ x_{4}\left(t\right) & =\text{rect}\left(\frac{t}{T}\right)\cdot \exp\left(j\pi k_{r}t^{2}\right)+\text{rect}\left(\frac{t-T}{T}\right)\cdot \exp\left(j\pi k_{r}t^{2}+\frac{3j\pi }{2}\right)\\ & +\text{rect}\left(\frac{t-2T}{T}\right)\cdot \exp\left(j\pi k_{r}t^{2}+j\pi \right)+\text{rect}\left(\frac{t-3T}{T}\right)\cdot \exp\left(j\pi k_{r}t^{2}+\frac{j\pi }{2}\right) \end{array}$$

where rect(·) represents the rectangular window function and the pulse width of the transmitted signal is 4*T*. Transform (4) into frequency domain and we get:

(5) $$\begin{array}{cc} X_{1}\left(4k+1\right) & =\mathrm{DFT}\left[\exp\left(\;j\pi k_{r}{\left(\frac{k-1}{F_{s}}\right)}^{2}\right)\right],X_{1}\left(p|p\neq 4k+1\right)=0\\ X_{2}\left(4k+2\right) & =\text{DFT}\left[\exp\left(\;j\pi k_{r}{\left(\frac{k-1}{F_{s}}\right)}^{2}\right)\cdot \exp\left(-j\frac{2\pi }{4N\left(k-1\right)}\right)\right],X_{2}\left(p|p\neq 4k+2\right)=0\\ X_{3}\left(4k+3\right) & =\text{DFT}\left[\exp\left(\;j\pi k_{r}{\left(\frac{k-1}{F_{s}}\right)}^{2}\right)\cdot \exp\left(-j\frac{2\pi }{4N}\cdot 2\left(k-1\right)\right)\right],X_{3}\left(p|p\neq 4k+3\right)=0\\ X_{4}\left(4k+4\right) & =\text{DFT}\left[\exp\left(\;j\pi k_{r}{\left(\frac{k-1}{F_{s}}\right)}^{2}\right)\cdot \exp\left(-j\frac{2\pi }{4N}\cdot 3\left(k-1\right)\right)\right],X_{4}\left(p|p\neq 4k+4\right)=0 \end{array}$$

for k=0,1,2,…,N−1, p=1,2,…,4N, the discrete Fourier transform (DFT) length is N and $N=F_{s}\cdot T$. Here $F_{s}$ denotes the sampling rate.

It can be known from the expression in frequency domain that the four signals are completely orthogonal in frequency domain, and when the information of the corresponding frequency points is extracted during demodulation, the corresponding transmitted signals can be completely recovered.

When the signal is received, assuming that the sampling rate is $F_{q}$, the number of sampling points is $N_{m}$, the condition of extracting at the original frequency point needs to be satisfied. This also means that $\frac{F_{s}}{4N}$ must be an integer multiple of $\frac{F_{q}}{N_{m}}$. If this condition is not satisfied, the echo needs to be filled with zeros in time domain to meet this requirement. Suppose $K=\frac{F_{s}}{4N}\cdot \frac{N_{m}}{F_{q}}$, the original signal can be restored by performing K-times extraction in frequency domain.

**Figure 3:**
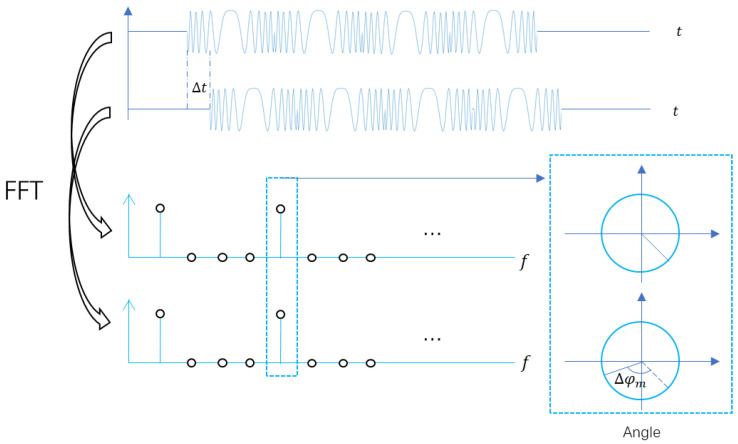
Relationship between phase shift and time delay.

The position of the target is determined by a radar based on the peak of time domain of the signal after pulse compression. In the case where the form of the signal is determined, the cyclic shift of the signal in time domain is equal to a phase shift in frequency domain. As shown in Figure 3.

The relationship between them is as following:

(6) $$\Delta \varphi_{m}=\frac{2\pi }{N_{m}}\cdot \Delta t\cdot F_{q}\cdot m=\frac{2\pi m\Delta tF_{q}}{N_{m}},m=0,1,2,\ldots ,N_{m}-1$$

where Δt denotes the time delay, m denotes the position of corresponding frequency after DFT and $\Delta \varphi_{m}$ denotes the phase shift of the *m*-th frequency point.

When extracting in frequency domain, the interval between adjacent frequency points is $\Delta f_{2}=\frac{KF_{q}}{N_{m}}$. The number of sampling points is $M_{2}=\frac{N_{m}}{K}$. The phase shift is:

(7) $$\Delta \varphi_{k}=\Delta \varphi_{Km}=\frac{2\pi }{N_{m}}\cdot \Delta t\cdot F_{q}\cdot K\cdot m=\frac{2\pi mK\Delta tF_{q}}{N_{m}},\;k=0,1,2,\ldots ,\frac{N_{m}}{K}-1$$

So

(8) $$\Delta \varphi_{k}\left(\Delta t+\frac{N_{m}}{F_{q}\cdot K}\right)=\frac{2\pi mK\left(\Delta t+\frac{N_{m}}{F_{q}\cdot K}\right)F_{q}}{N_{m}}=\frac{2\pi mK\Delta tF_{q}}{N_{m}}+\frac{2\pi KmF_{q}}{N_{m}}\frac{N_{m}}{F_{q}\cdot K}=\Delta \varphi_{k}\left(\Delta t\right)+2\pi m$$

It means that, if the time difference between the arrival of the echoes of two targets is $\tau =\frac{N_{m}}{F_{q}K}$, then the targets cannot be separated. Because they have no difference in frequency domain after extracting.

**Figure 4:**
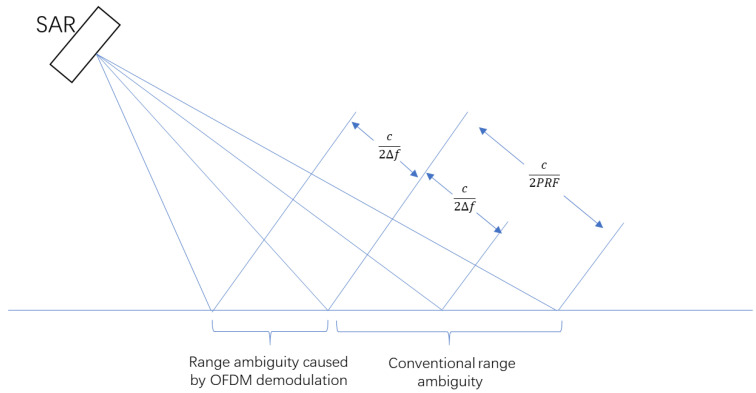
Geometric sketch of range ambiguity.

Assume that the transmitted OFDM chirp signal has subcarrier frequency interval of Δf, then $K=\frac{N_{m}\Delta f}{F_{q}}$, so the maximum time interval of targets’ echoes is $\Delta t_{max}=\frac{N_{m}}{F_{q}K}=\frac{1}{\Delta f}$. That is, the swath width cannot be greater than $\frac{c_{0}}{2\Delta f}$, where $c_{0}$ is the speed of light. In practical applications, it is necessary to convert the slope distance to the ground distance to calculate the specific the swath width. Exceeding this limit will cause range ambiguity, as shown in Figure 4. The above analysis is applicable to any multiple orthogonal signals.

For the limitation of range ambiguity, DBF can be used to limit the width to maximum unambiguous swath width. However, this method uses a small antenna to send a wide beam. The transmission of a wide beam needs to reduce the aperture of the antenna, which reduces the gain of antenna and cannot meet the requirement of signal-to-noise ratio. If a wide beam is not used, the swath width of each imaging strip is seriously limited. If a large mapping width is required, it can only be achieved by inter-pulse scanning or multiple flights. Inter-pulse scanning requires more azimuth PRF, and multiple flight requires more time cost, both of them cannot meet the needs of high-resolution wide-swath mapping.

## 3. Simultaneous Digital Beamforming of Transceiver and Receiver

**Figure 5:**
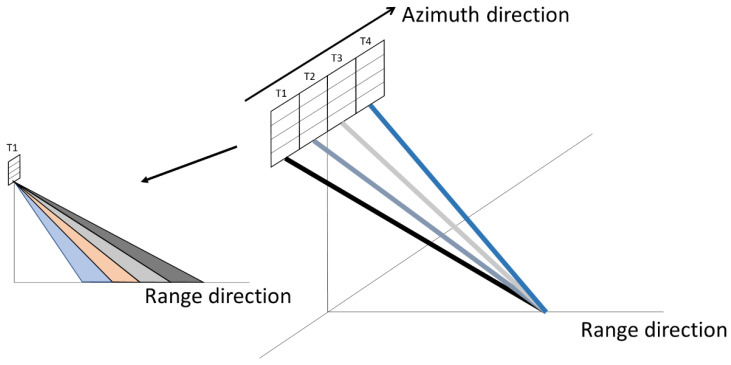
Schematic diagram of the proposed system.

This paper designs a MIMO-SAR system using simultaneous digital beam forming of both transceiver and receiver to avoid range ambiguity and increase the energy efficiency. For multiple transmitting antennas in azimuth, the orthogonality is guaranteed by the generator matrix of the OFDM chirp signal. For the transmitting antenna in each azimuth, the orthogonality between multiple sub-bands in the range direction is guaranteed by frequency diversity and DBF. The schematic diagram of each azimuth antenna in the range direction to achieve multiple sub-bands mapping is shown in Figure 5.

**Figure 6:**
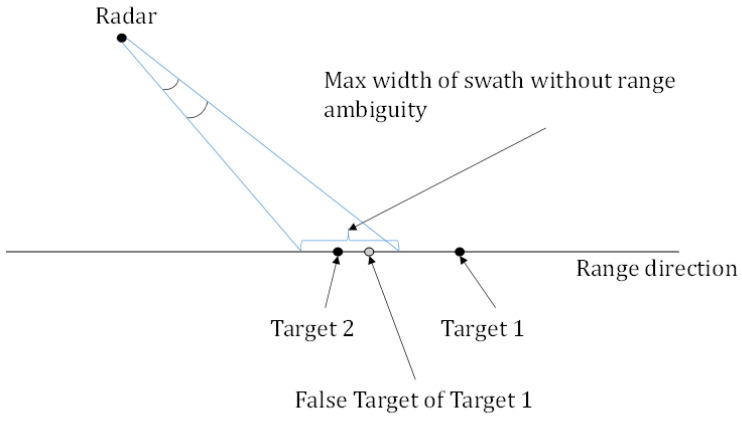
Principle of suppressing the range ambiguity.

Multiple sub-beams are transmitted by digital beam forming in range, each sub-beam adopts signals of different frequencies, and satisfies the demodulation limitation of the OFDM chirp signal. The principle of suppressing the range ambiguity is shown in Figure 6. The echo of false target is beyond the maximum unambiguous distance. The target in each sub-band can be retained by the spatial filtering and the frequency filtering. The targets other than the sub-band will be suppressed after the spatial and frequency filtering. This scheme can meet the requirements of HRWS remote sensing without range ambiguity.

The signals can be transmitted by a phased array antenna. The following will discuss how to implement this method with 4 sub-bands as an example.

**Figure 7:**
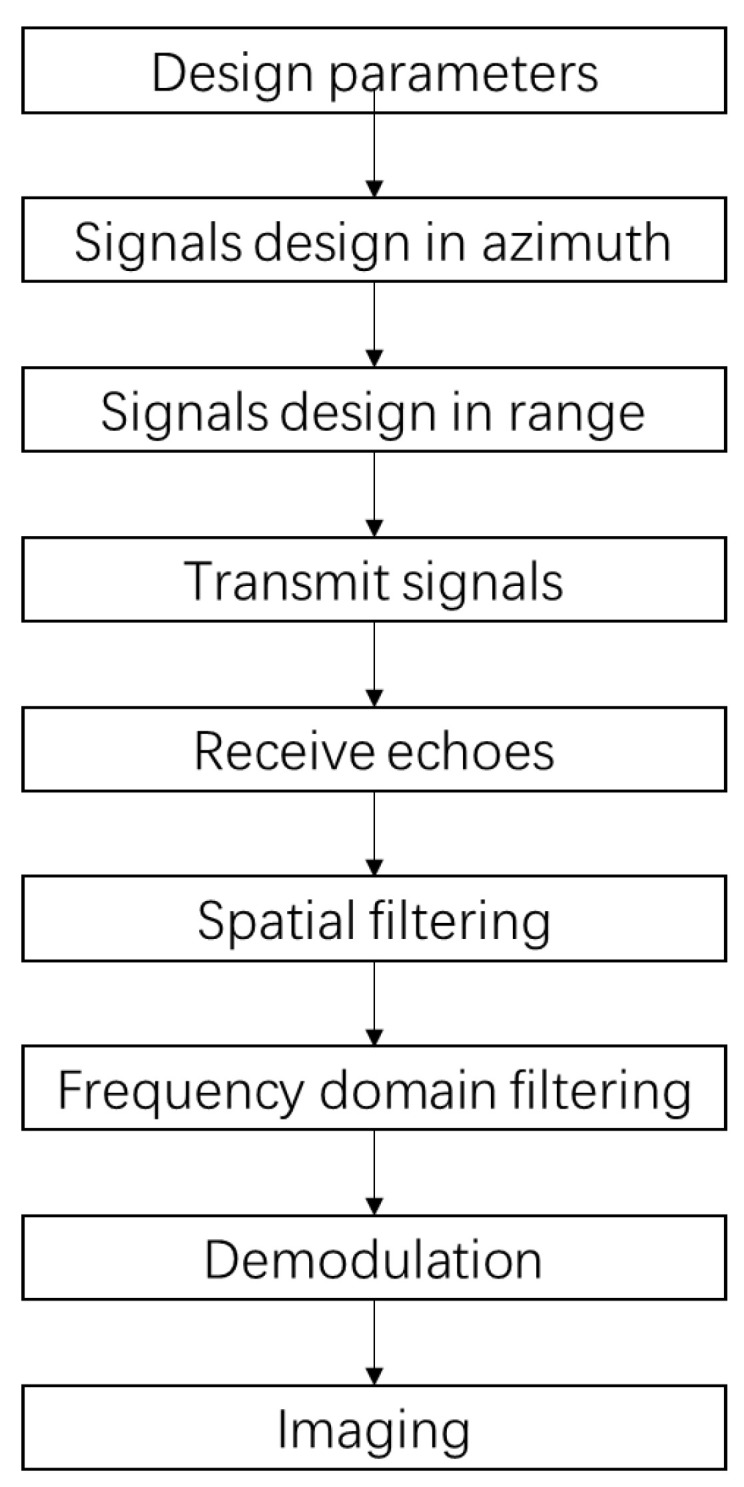
Diagram of the MIMO-SAR system designed in this paper.

Figure 7 shows the imaging process of the MIMO-SAR system mentioned in this paper.

The steps of the MIMO-SAR imaging method proposed in this paper based on multi-dimensional waveform coded signals and simultaneous digital beamforming of both transceiver and receiver are:

Step 1: Set the relevant system parameters. M is the number of azimuth transmitting antennas; $N_{r}$ is the number of range sub-bands; and P is the number of receiving antennas.
Step 2: According to the number of azimuth transmitting antennas, design M orthogonal multi-dimensional waveform coding signals. These signals can be expressed by $S_{m},m=1,2,\ldots ,M$. The OFDM-chirp signal forming matrix is expressed as (2).
Step 3: According to the number of the sub-bands in the range, design the transmission waveform in the range direction of each array element.

The beamforming can be implemented in digital domain or in analog domain. In analog domain, multiple beams can be realized through a passive power synthesis network. In digital domain, multiple signals with different phase offsets can be generated by a digital signal generator. In the system designed in this paper, each orthogonal signal in different transmit antenna is composed of chirp signal with different phase modulation. Chirp signal has constant modulus. Therefore, the nonlinear characteristics of the power amplifier will not affect the characteristics of the signals. Multiple beams can be realized through a passive power synthesis network. In this case, the characteristics of the signal will not be changed. If the signal is synthesized directly in digital domain, it is necessary for the system to ensure a high linearity. The following will introduce the simultaneous digital beamforming technology in digital domain in detail.

**Figure 8:**
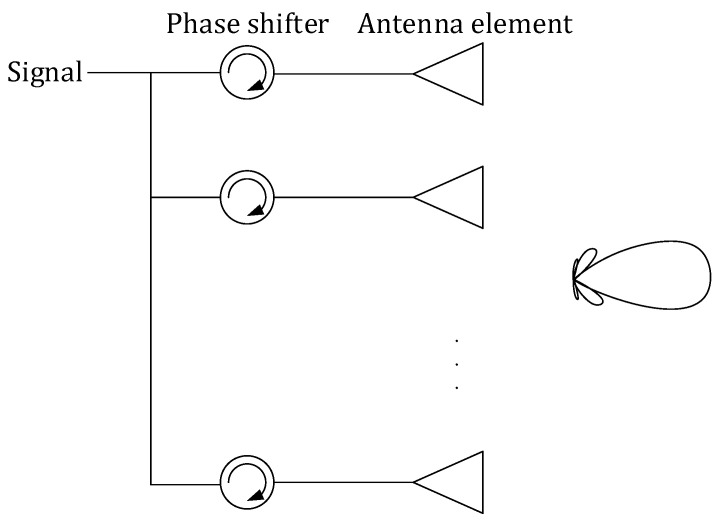
Schematic diagram of the traditional beamforming.

**Figure 9:**
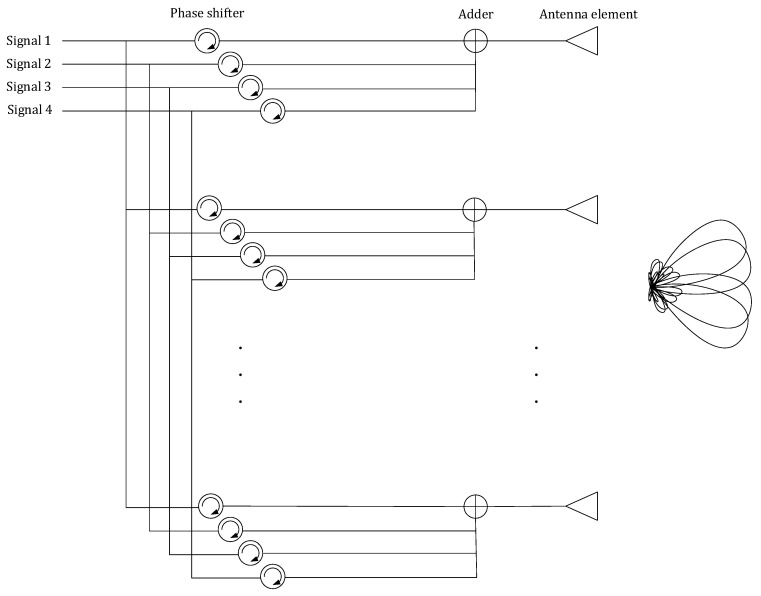
Schematic diagram of the proposed system in elevation direction.

As shown in Figure 8, traditional beamforming uses different phases of signals in different antenna elements to achieve spatial beam pointing. The method of transmitting signals mentioned in this paper is shown in Figure 9. Each antenna element synthesizes signals of different directions and transmits them. It is worth mentioning that if these signals have the same frequency, there will be crosstalk between different beams. In order to avoid this problem, signals with different directions should be distinguished in frequency domain.

When $N_{r}=4$, their center frequencies are $f_{i},i=1,2,\ldots ,N_{r}$, the corresponding wavelength is $\lambda_{i},i=1,2,\ldots ,N_{r}$, the number of array elements along the elevation direction is *L*, the spacing of the array elements is d and the corresponding orthogonal waveform encoding signal is $S_{mi}$. $S_{mi}$ denotes the signal with the center frequency of $f_{i}$ and transmitted by the m-th transmitting antenna. $\theta_{c,i}$ denotes the angle between the center angle of ith sub-beam and the normal of the array antenna. Then the mapping swath in range direction is divided into several sub-bands. The division can use the following ways:

The transmitted signal of the range to each array element is $s_{l}$, l=1,2,…,L, and we can get:

(9) $$s_{l}=\overset{4}{\underset{i=1}{\sum }}S_{mi}\cdot \exp\left(\frac{j\left(l-1\right)\left(2\pi d\sin\theta_{c,i}\right)}{\lambda_{i}}\right)$$

The synthesized normalized antenna pattern can be expressed as:

(10) $$G_{i}\left(\theta \right)=\frac{\sin^{2}\left[L\pi \left(\frac{d}{\lambda_{i}}\right)\left(\sin\theta -\sin\theta_{c,i}\right)\right]}{L^{2}\sin^{2}\left[\pi \left(\frac{d}{\lambda_{i}}\right)\left(\sin\theta -\sin\theta_{c,i}\right)\right]},i=1,2,3,4$$

where $G_{i}\left(\theta \right)$ denotes the antenna pattern of the ith beam along the elevation direction.

Step 4: The signal is transmitted through the transmitting module.
Step 5: Receive the signal through the signal receiving module, then sample the signal.
Step 6: Separate the reflected echoes with different range to different subswath through spatial filtering.

The spatial filtering method is to perform weight processing on the signals received by *L* array elements in range, so as to obtain signals corresponding to the beam pointing. The corresponding weighting matrix can be expressed as:

(11) $$V_{i}=\left[\begin{array}{ccccc} 1 & e^{j\frac{2\pi d\sin\theta_{c,i}}{\lambda_{i}}} & e^{j2\frac{2\pi d\sin\theta_{c,i}}{\lambda_{i}}} & \ldots & e^{j\left(L-1\right)\frac{2\pi d\sin\theta_{c,i}}{\lambda_{i}}} \end{array}\right]$$

Assuming that the received signal of each array element is $sr_{m},m=1,2,\ldots ,L$ the signal after filtering in spatial domain is $S_{ri}$, we can get:

(12) $$S_{ri}=\left[\begin{array}{cccc} sr_{1} & sr_{2} & \ldots & sr_{L} \end{array}\right]V_{i}{}^{T}$$

Step 7: After filtering in frequency domain, the reflected echoes of different subswath are further separated to improve the isolation between the signals of the respective subswath.

The filtering in frequency domain is performed on the signal after filtering in spatial domain, and the obtained signal is $SR_{i}$:

(13) $$SR_{i}=\mathrm{IFFT}\left(\mathrm{FFT}\left(S_{ri}\right)\cdot H_{i}\right)$$

where $H_{i}$ is a bandpass filter with a center frequency of $f_{i}$. Its band-pass range should be slightly larger than the signal’s bandwidth without aliasing. IFFT (·) is inverse fast Fourier transform. FFT (·) is fast Fourier transform.

Step 8: Demodulate each of the separated subswath signal to obtain the receiving echo corresponding to the transmitting antenna in each azimuth. The transmitted signals in frequency domain is expressed as (3).

After FFT is performed on the received echoes, the corresponding frequency points in frequency domain are extracted to demodulate the corresponding azimuth orthogonal signal.

Step 9: Use traditional MIMO-SAR imaging algorithm to process the echoes after steps 6–8.

## 4. Simulation Results

### 4.1. Simulation Results of the Proposed System

**Figure 10:**
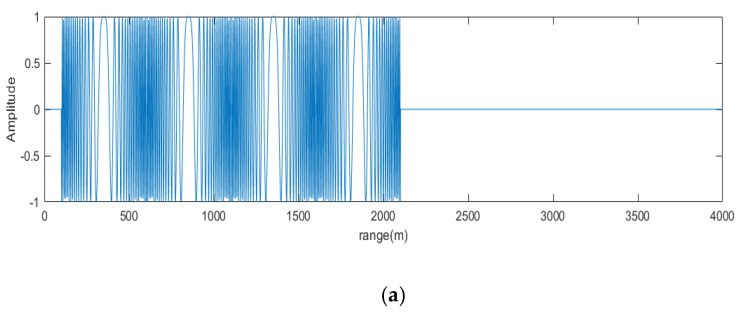

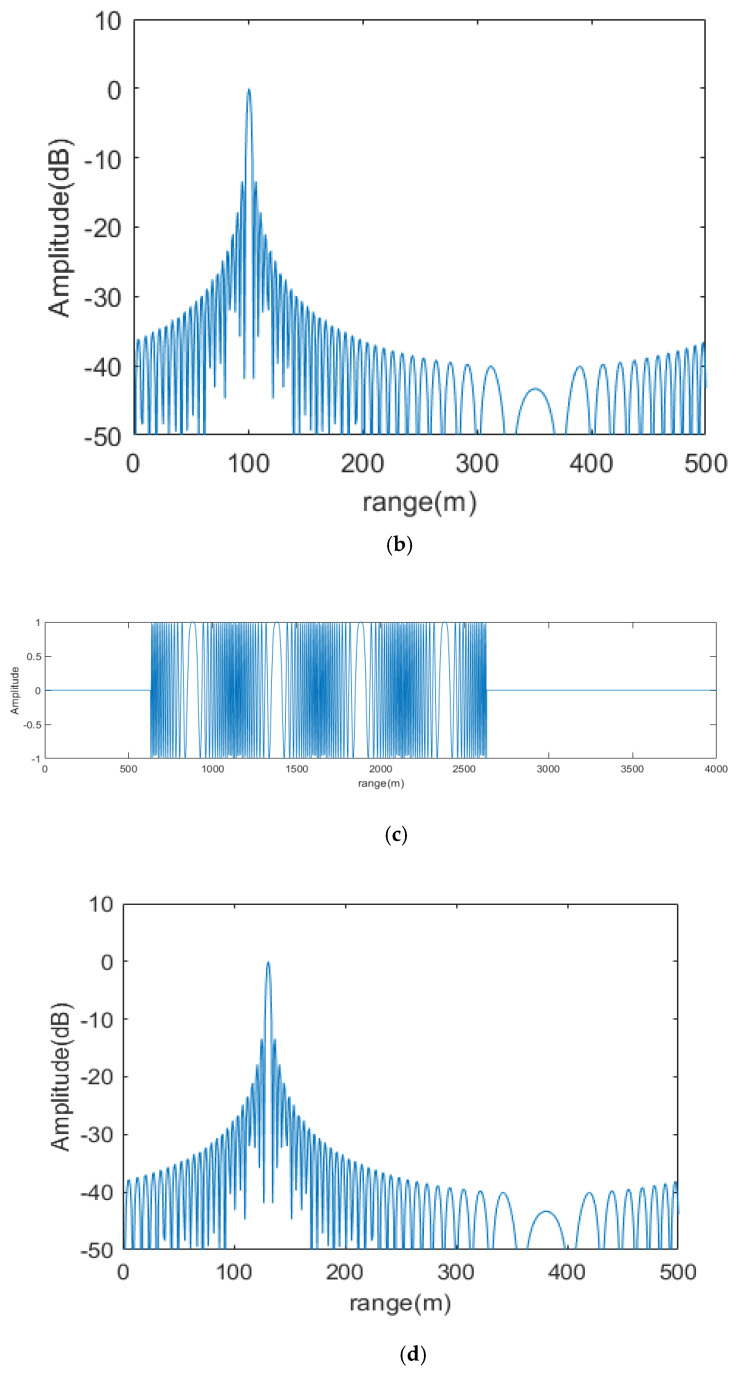

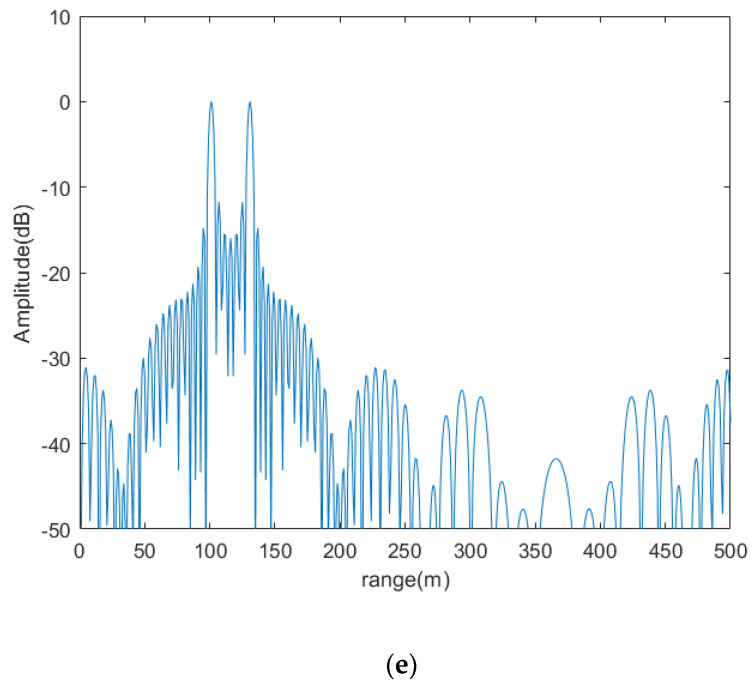
Simulation results. (**a**) echo of target 1. (**b**) demodulation results of target 1. (**c**). echo of target 2. (**d**) demodulation results of target 2. (**e**) demodulation results of mixed echo.

In order to verify the effectiveness of the proposed system, some simulations were designed. Suppose there are two targets, the distance from the reference point of target one is 100 m and 630 m for target two. Value of parameters is shown in Table 1. As can be seen from Figure 10, the distance between the two targets after demodulation is 30m. Aliasing occurs because the distance of two targets is greater than the maximum unambiguous distance.

**Figure 11:**
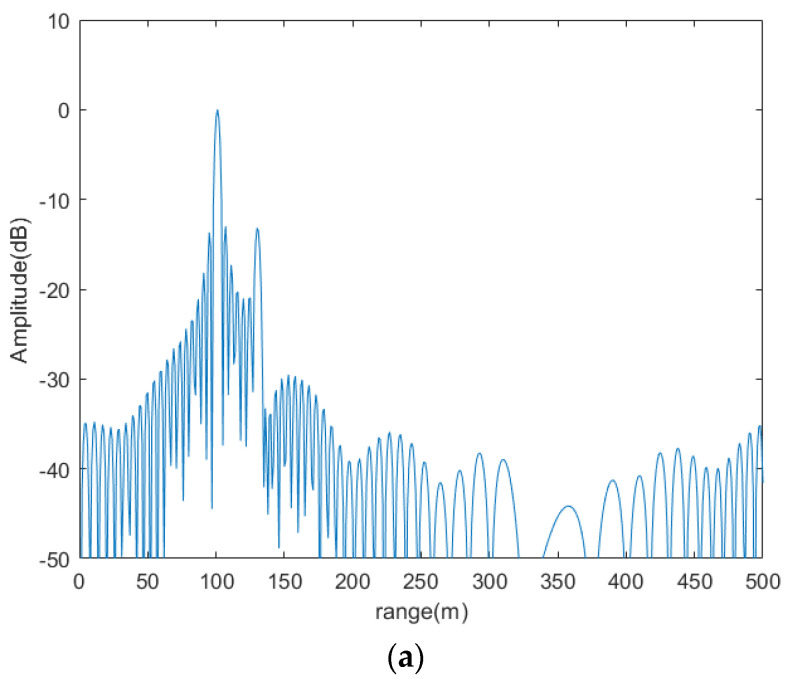

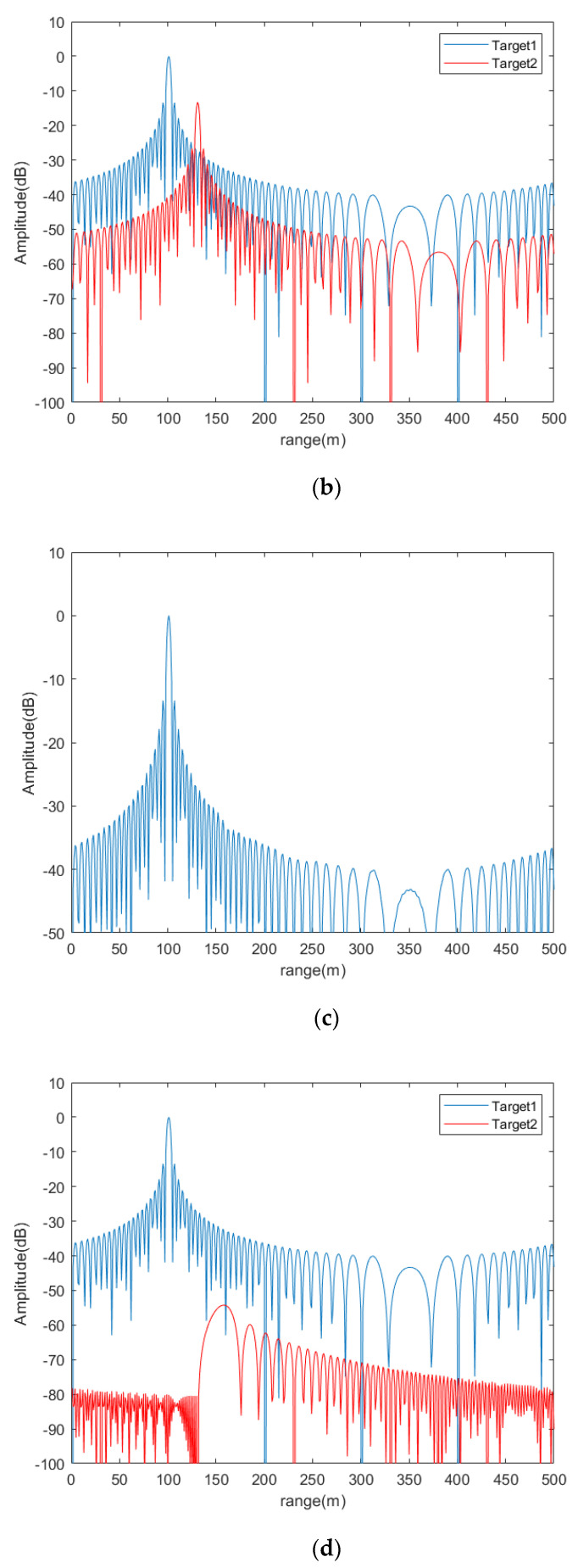
Ability of suppressing the range ambiguity. (**a**) demodulation results of mixed echo with digital beam forming (DBF) in receiving. (**b**) demodulation results of each target with DBF in receiving. (**c**) demodulation result of mixed echo with proposed method. (**d**) demodulation results of each target with proposed method.

Figure 11 shows the ability of the traditional method and the proposed method to suppress range ambiguity. As can be seen from Figure 11b, the ability of the traditional method to suppress range ambiguity is −13.3 dB, and the ability of the proposed method to suppress range ambiguity is −54.2 dB from Figure 11d.

**Figure 12:**
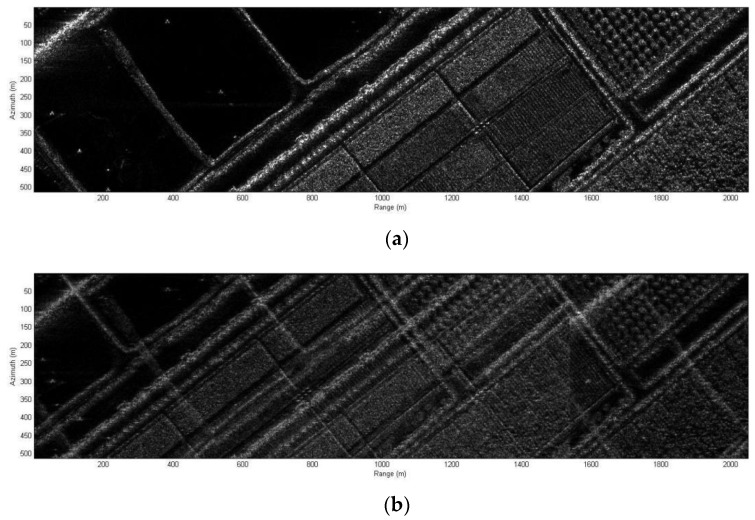

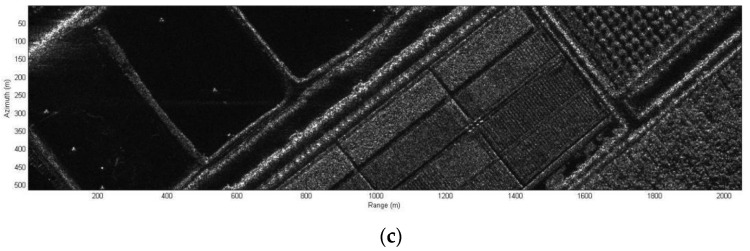
Simulation results. (**a**) origin scene. (**b**) reconstructed image with the range ambiguity of OFDM chirp signal. (**c**) reconstructed image with the proposed system.

Simulation results of real-scene are as shown in Figure 12. Value of parameters is shown in Table 2 and Table 3.

It can be seen from Figure 12b, the image quality will be seriously limited because of the range ambiguity without DBF. Figure 12c shows a result with the proposed system. The range ambiguity is suppressed by dividing the swath into four subswaths whose width is less than the maximum swath width without range ambiguity. Through the above real-scene simulations, we can conclude from that the novel MIMO-SAR systems has been validated.

In the above simulation, we only considered the effect of range ambiguity suppression when the uniform linear array is not weighted. If the receiver antenna elevation pattern was tapered by a Dolph–Chebyshev window, the range ambiguity can be further suppressed. It is worth mentioning that, this does not affect the range ambiguity suppression ability of the proposed scheme, since DBF is also used on the transmitter. In Section 4.2, we will further analyze the method while the antenna elevation pattern is tapered by a Dolph–Chebyshev window.

### 4.2. Performance Comparison

The above simulation shows the effectiveness of the proposed scheme. In order to further illustrate the advantages of the proposed scheme, we compare our scheme with the scheme in [6]. Except for the height of the transmitting antenna and the total bandwidth, other parameters are set in accordance with [6], as shown in Table 4. The height of the transmitting antenna is increased to enable DBF at the transmitting end. Increasing the total bandwidth is to reduce the interference between different beams.

The performance of the MIMO-SAR system is compared with [6] in terms of the noise equivalent sigma zero (NESZ) and range-ambiguity-to-signal ratio (RASR).

NESZ is a parameter which indicates the system radiometric sensitivity. It is defined as the backscattering coefficient corresponding to an SNR that is equal to unity. In MIMO-SAR, NESZ is as follows:

(14) $$\mathrm{NESZ}=\frac{256\cdot {\left(\pi \cdot R\right)}^{3}\cdot k_{b}\cdot T_{noise}\cdot L_{loss}\cdot V\cdot B\cdot \sin\theta }{P_{m}\cdot G_{t}\left(\theta \right)\cdot G_{r}\left(\theta \right)\cdot \lambda^{3}\cdot c_{0}}$$

where R is the slant range between the radar sensor and the target, $k_{b}$ is the Boltzmann’s constant of $1.3807\times 10^{-23}J\cdot K^{-1}$, $P_{m}$ is the average transmit power, $L_{loss}$ is the loss of antenna feed networks, $G_{t}$ and $G_{r}$ are the gain patterns of the transmit array and the receive array, respectively, θ denotes the elevation angle, λ is the wavelength, $T_{noise}$ is the equivalent system noise temperature and V is velocity of the platform in the azimuth direction.

**Figure 13:**
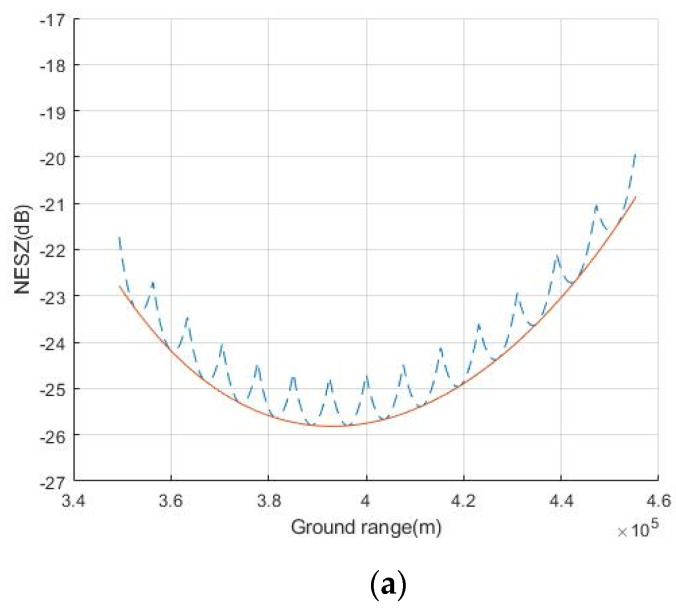

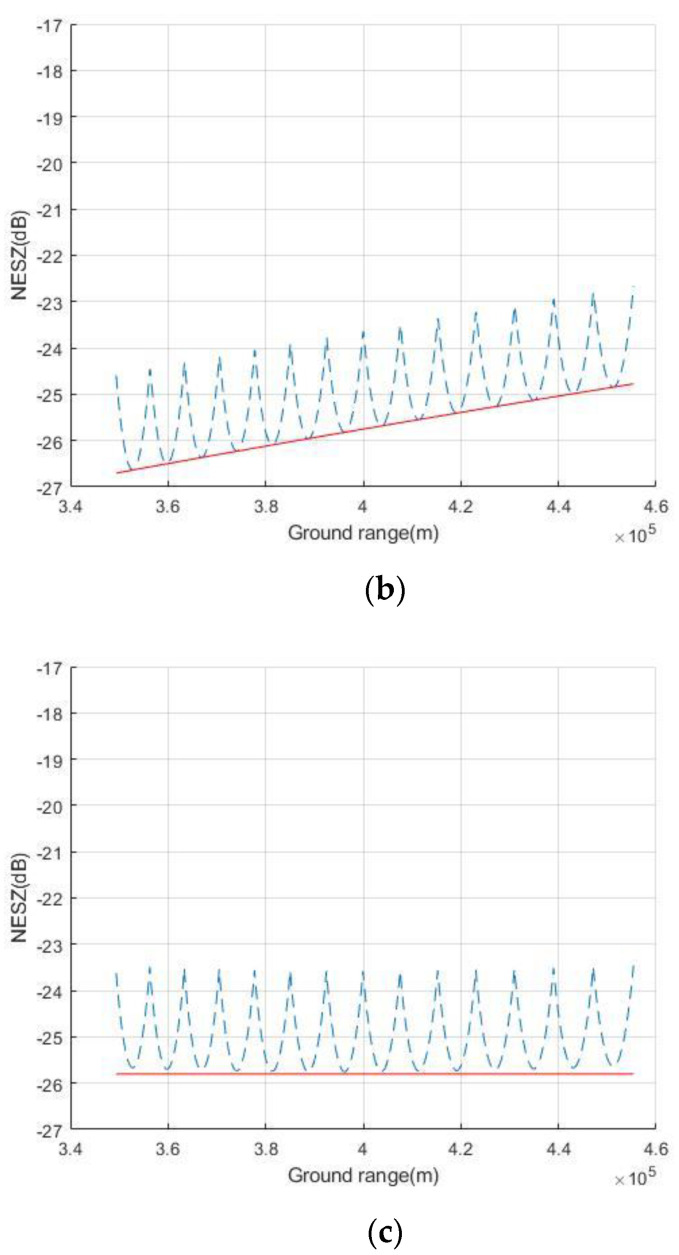
Noise equivalent sigma zero (NESZ) of the system. (**a**) NESZ of the system in [6]. (**b**) NESZ of the system proposed in this paper when the energy is evenly distributed to each subswath. (**c**) NESZ of the system proposed in this paper when the energy is distributed to each subswath as needed.

As can be seen from Table 4, compared with [6], the proposed scheme has a larger transmitting antenna, which provides a higher transmitting antenna gain. Although transmitting multiple beams will reduce the power of each beam to offset these gains, the proposed scheme can better concentrate the energy in the mapping swath. Figure 13a shows NESZ of the system in [6]. The dashed–dotted line denotes the NESZ of each subswath, and the solid line highlights the coherent combination of the subswath data. As can be seen from Figure 13b, the system proposed in this paper achieves better performance of NESZ. At the same time, due to the higher degree of freedom of the transmitting array, more energy can be allocated to the farther subswath. After redistribution of energy, the NESZ is shown in Figure 13c. It is worth mentioning that this scheme will have a broader application prospect in a larger swath width. When the swath width increases, the distance difference between the far end and the near end will be greater, resulting in the obvious change of NESZ. The NESZ of the proposed system can achieve below −23 dB.

The RASR is used to indicate the range ambiguity effect. For the MIMO-SAR system using OFDM chirp signals, this parameter is defined as follows [6]:

(15) $$\text{RASR}=\frac{R_{0}^{3}\cdot \sin\left(\theta_{in}\right)}{{\left|C_{2way}\left(\theta \right)\right|}^{2}}\cdot \overset{N_{far}}{\underset{\begin{array}{c} p\neq 0\\ p=N_{near} \end{array}}{\sum }}\frac{{\left|C_{2way}\left(\theta_{p}\right)\right|}^{2}}{R_{0,p}^{3}\cdot \sin\left(\theta_{in,p}\right)}+\frac{R_{0}^{3}\cdot \sin\left(\theta_{in}\right)}{{\left|C_{2way}\left(\theta \right)\right|}^{2}}\cdot \overset{M_{far}}{\underset{\begin{array}{c} q\neq 0\\ q=M_{near} \end{array}}{\sum }}\frac{{\left|C_{2way}\left(\theta_{q}\right)\right|}^{2}}{R_{0,q}^{3}\cdot \sin\left(\theta_{in,q}\right)}$$

where $\theta_{in}$ denotes the incidence angle and $C_{2way}$ is the two-way antenna pattern. The subscript p indicates the ambiguous signal order coursed by the conventional range ambiguity. The subscript q indicates the ambiguous signal order coursed by the OFDM demodulation. $N_{naer}$, $M_{naer}$ and $N_{far}$, $M_{far}$ give the number of pulses considered in the calculation in both near and far ranges, respectively.$\;R_{o}$ is the original distance to a target, and $R_{o,p}$ is the slant range between the radar sensor and the position of the pth ambiguous signal source, corresponding to an elevation angle $\theta_{p}$ and incidence angle $\theta_{in,p}$. $R_{o,q}$ is the slant range between the radar sensor and the position of the qth ambiguous signal which caused by OFDM demodulation, corresponding to an elevation angle $\theta_{q}$ and incidence angle $\theta_{in,q}$. By adjusting the appropriate receiving window, the range ambiguity caused by OFDM demodulation can be further suppressed.

**Figure 14:**
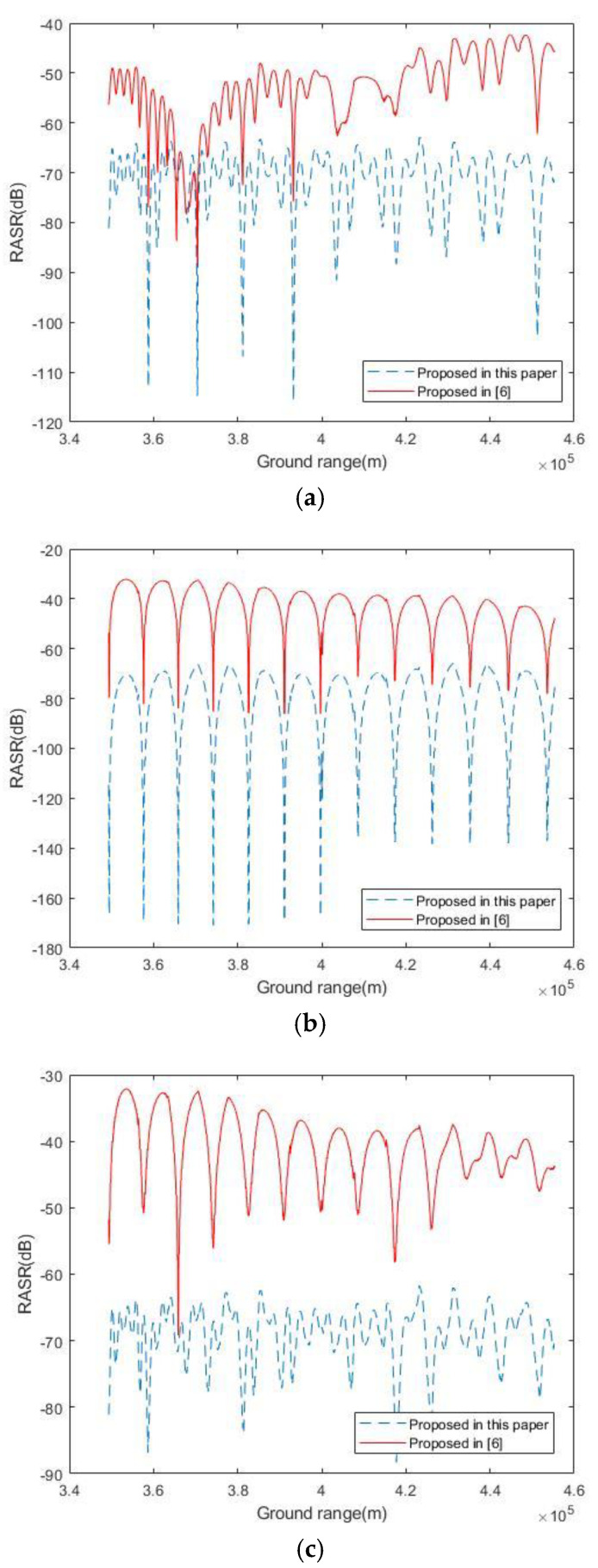
Computed range-ambiguity-to-signal ratio (RASR) over the whole swath: (**a**) conventional RASR part. (**b**) RASR caused by the OFDM demodulation. (**c**) the final RASR. The antenna elevation pattern was tapered by a Dolph–Chebyshev window for a sidelobe level of −35 dB.

As can be seen from Figure 14a, the range ambiguities are suppressed in the antenna pattern by 40 dB in [6]. Benefit by the spatial degree of freedom provided by the transmitting antenna array, the traditional RASR of the system we propose can reach below 60 dB. Similarly, in Figure 14b, the proposed system has better performance for RASR caused by OFDM demodulation. We also get a better RASR than [6] in the final RASR, which is shown in Figure 14c. The antenna elevation pattern was tapered by a Dolph–Chebyshev window for a sidelobe level of −35 dB.

Table 5 gives a performance comparison of the proposed MIMO-SAR system in this paper with the system proposed by Kim in [6]. Obviously, lower NESZ and more effective suppression of range ambiguities are achieved from the proposed MIMO-SAR system.

## 5. Discussion

In this paper, firstly, the two-channels orthogonal OFDM chirp signals are extended to M-channels, where M is any integer. Then the cause of range ambiguity of the OFDM chirp signal is detailed. For the limitation of range ambiguity, the traditional method uses DBF to limit the width to maximum unambiguous swath width. An innovative MIMO-SAR system uses OFDM chirp signal which can not only solve the problem of range ambiguous but also increase antenna gain is designed in this paper. For multiple transmitting antennas in azimuth, the orthogonality is guaranteed by OFDM chirp signals. For the transmitting antenna in each azimuth, the orthogonality between multiple sub-swaths in the elevation direction is guaranteed by frequency diversity and DBF. Due to the increase of spatial degrees of freedom, the proposed scheme has lower NESZ and RASR. The swath width is limited by PRF in traditional systems, the system proposed in this paper may have the potential to break through this limitation, since the swath is divided into several subswath. Simulation results demonstrate the effectiveness of the proposed method.

OFDM chirp signal is sensitive to Doppler-shift, it is a disadvantage. Some scholars have studied how to compensate for the Doppler-shift [28]. However, for complex multi-antenna channels, the method has limitations. Limited to the length of this paper, we will describe in detail how to compensate for this in the future. Our future work also includes exploiting the advantages of the scheme by using a practical airborne MIMO-SAR system in HRWS remote sensing.

## Figures and Tables

**Table 1:** Parameters Used in Figure 10 and Figure 11.

Parameters	Value
bandwidth	150 MHz
swath width	2000 m
pulse subcarrier spacing	300 kHz
maximum swath width without range ambiguity	500 m

**Table 2:** Parameters Used in Figure 12b.

Parameters	Value
bandwidth	150 MHz
swath width	2000 m
pulse subcarrier spacing	300 kHz
maximum swath width without range ambiguity	500 m
center frequency	5.4 GHz

**Table 3:** Parameters Used in Figure 12c.

Parameters	Value
bandwidth	150 MHz
swath width	2000 m
pulse subcarrier spacing	300 kHz
maximum swath width without range ambiguity	500 m
center frequency of sub-beam 1	5.2 GHz
center frequency of sub-beam 2	5.4 GHz
center frequency of sub-beam 3	5.6 GHz
center frequency of sub-beam 4	5.8 GHz

**Table 4:** System parameter.

Parameters		Values
Geometry	swath width	~100 km
	Number of subswaths	14
	orbit height	560 km
	velocity	7560 m/s
	spatial resolution	1 m × 1.5 m
		(range × azimuth)
	center frequency	9.65 GHz
Transmit array	length	2.5 m
	total height	3.5 m
	gain	51 dBi
	Number of antennas	2
Receive array	total length	9.6 m
	total height	3.5 m
	gain	56 dBi
	azimuth panel	6
	elements in elevation	42
HRWS system	peak power	5 kW
	average power	max. 1.208 kW
	bandwidth per subswath	250 MHz
	pulse duration	150 μsec
	duty cycle	24.10%
	noise figure	3.75 dB
	loss	3 dB
	pulse subcarrier spacing	6.67 kHz
	pulse repeat frequency	1.6 kHz

**Table 5:** Comparison of the system.

Different Systems	NESZ	RASR
system proposed in [6]	<−20 dB	<−30 dB
system proposed in this paper	<−23 dB	<−60 dB
